# Long-term follow-up of repair-like replacement of mitral valve using autologous pericardium

**DOI:** 10.1016/j.xjtc.2024.02.020

**Published:** 2024-03-06

**Authors:** Tomoki Shimokawa, Hitoshi Kasegawa, Minoru Tabata, Toshihiro Fukui, Hajime Kin, Atsushi Shimizu, Tomoya Uchimuro, Kenta Zaikokuji, Schuichiro Takanashi

**Affiliations:** a Department of Cardiovascular Surgery, Sakakibara Heart Institute, Tokyo, Japan; b Department of Cardiovascular Surgery, Teikyo University, Tokyo, Japan; c Department of Cardiac Surgery, International University of Health and Welfare, Mita Hospital, Tokyo, Japan; d Department of Cardiovascular Surgery, Juntendo University, Tokyo, Japan; e Department of Cardiovascular Surgery, Kumamoto University, Kumamoto, Japan; f Department of Cardiovascular Surgery, Iwate University, Iwate, Japan; g Department of Cardiac Surgery, Kawasaki Saiwai Hospital, Kanagawa, Japan

**Keywords:** mitral valve repair, mitral valve replacement, stentless mitral valve, Normo valve, autologous pericardium

## Abstract

**Objectives:**

The present study assessed the late results of the operation, which consisted of the construction of a stentless mitral valve using autologous pericardium and valve implantation.

**Methods:**

Between 2011 and 2018, among 1617 consecutive patients who underwent mitral valve operation at our institution, 15 adult patients (0.9%) with unrepairable mitral valves who wished to avoid conventional mitral valve replacement underwent this operation. Ten patients (67%) had a history of valve repair. After discharge, patients were prospectively followed-up with a echocardiographic evaluation up to the end point. The mean follow-up term was 70.8 ± 42.5 months.

**Results:**

There were no hospital deaths or thromboembolic events and only 1 late noncardiac death. Intraoperative transesophageal echocardiography of all patients revealed no or trivial mitral regurgitation. Eight patients (53.3%) underwent redo valve replacement within 12 years. Except 1 late death, the postoperative course was divided into 3 groups depending on the occurrence of redo surgery, as follows: an early reoperation group (reoperation within 4 years; n = 4), a late reoperation group (reoperation after 4 years; n = 4), and a free from reoperation group (n = 6). The latest transthoracic echocardiographic examination performed 7.2 ± 2.9 years after the operation revealed the grade of mitral regurgitation to be none in 2 patients, mild in 2 patients, mild to moderate in 1 patients, and moderate in 1 patient in the free from reoperation group.

**Conclusions:**

Despite the high incidence of reoperation, Normo operation can be a viable option during valve replacement, especially for young patients.

## Graphical Abstract

Among 1617 consecutive patients who underwent mitral valve (MV) surgery at the Sakakibara Heart Institute between 2011 and 2018, 15 patients with unrepairable MV who strongly requested MV repair were enrolled in this study. Although the long-term echo findings were variable, perhaps depending on the quality of the autologous pericardium used, in patients without early progression of MR, echo findings were stable in the long-term.

**Figure:**
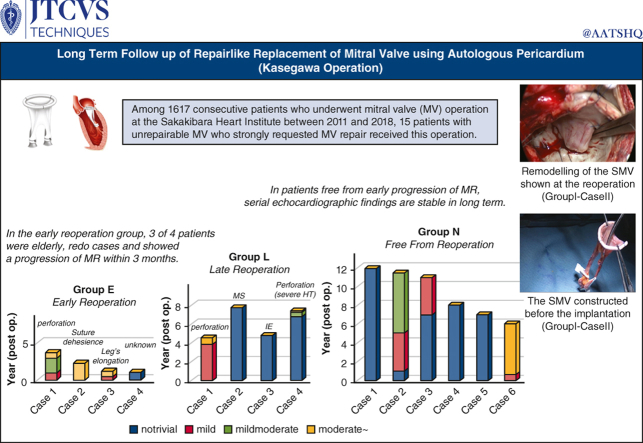

**Figure:**
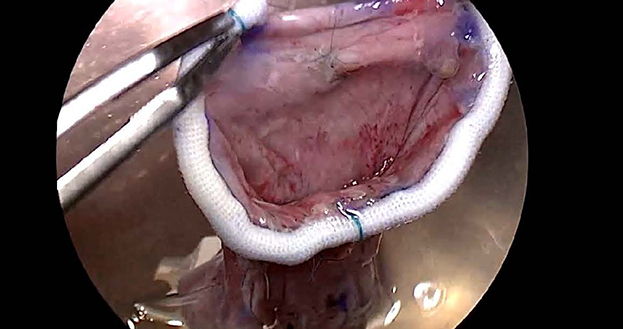
The Normo valve made from fresh nontreated autologous pericardium was constructed using a specially designed template before the institution of cardiopulmonary bypass.

**Central Message**

Normo operation is a viable option during valve replacement for young patients. A higher failure rate may occur with lower quality autologous pericardium in elderly and reoperative patients.

**Perspective**

Although echo follow-up demonstrated the variableness of the material, in patients free from the early progression of mitral regurgitation, echo findings were stable in the long-term. Normo operation can be a viable alternative to bioprosthesis replacement in select patients.

The incidence of mitral valve (MV) repair has been increasing^1^ since the end of the 20th century, accompanied by an improving trend in patients’ quality of life. However, MV repair for patients with complicated pathology remains challenging,^2^^,^^3^ and repair has not been attempted to avoid the risk of reoperation in many institutions. Furthermore, because the feasibility of successful redo repair is reported to be low,^4^ MV replacement (MVR) rather than MV repair is generally performed for recurrence of mitral regurgitation (MR) after complicated MV repair.

To overcome the disadvantages of the existing MVR techniques, Kasegawa and colleagues^5^ designed the 2-leaflet stentless mitral valve (SMV) they called the Normo valve in 2002 (Figure 1). The design concepts of the Normo valve, presented in Table 1, provide a deep coaptation area, and the anatomic features of this valve can ensure a high level of competency with good left ventricle function. The Normo operation consists of the construction of a SMV using fresh autologous pericardium (AP) before cardiopulmonary bypass and valve implantation. The hydrodynamic function of a valve made from bovine pericardium has been tested using a pulsatile MV simulator combined with echocardiography at the Center for Advanced Biomedical Sciences of Waseda University, Tokyo, Japan.^5^ After successful implantation for experimental animals, a meeting to explore the future development of SMVs involving 10 specialists in this field from Japan was held in 2010. The operative procedure was approved at the meeting.

**Figure 1:**
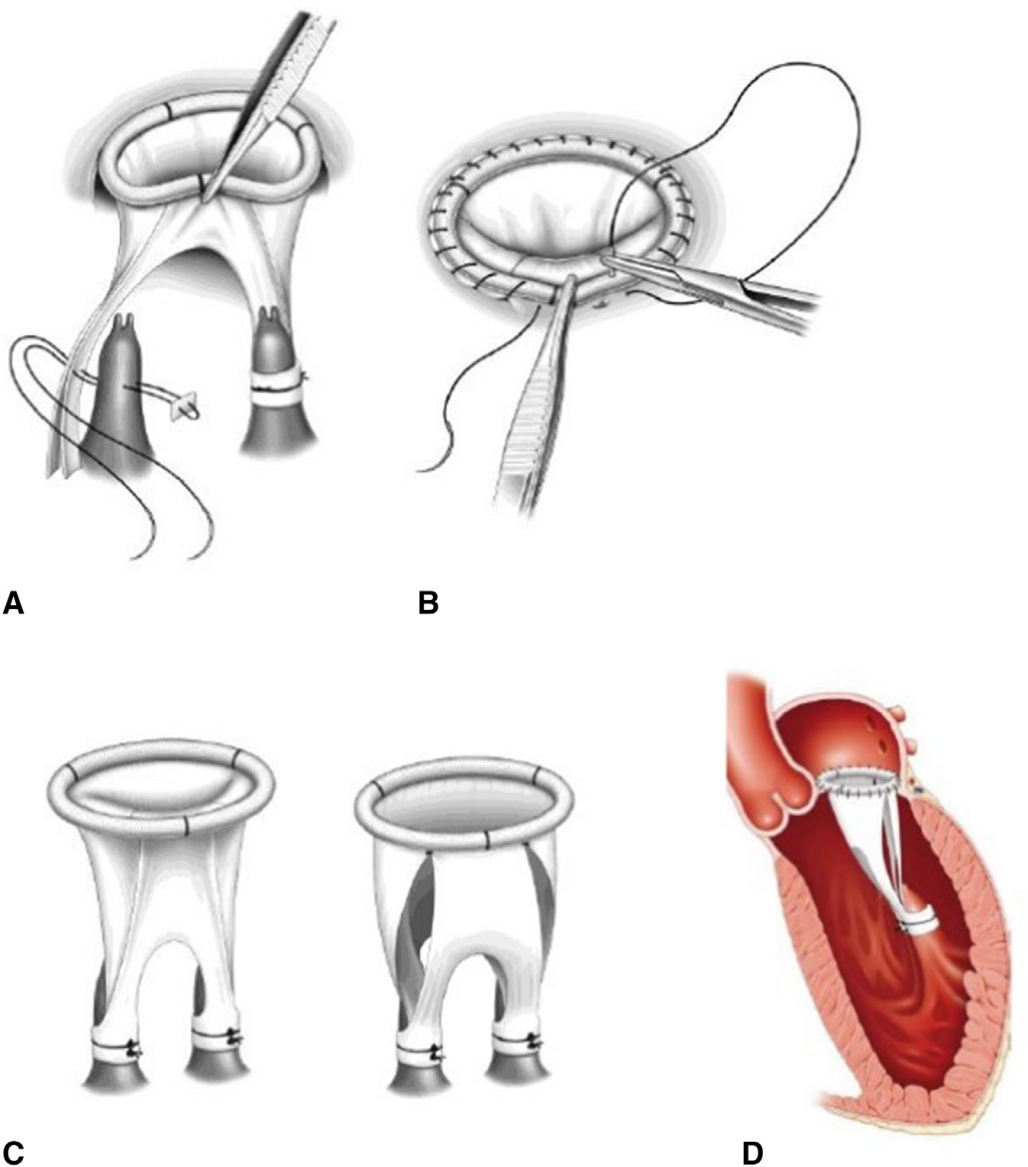
Normo operation. The Normo procedure consists of construction of the stentless mitral valve (*SMV*) and valve implantation. A, The fixation of the leg of the SMV to 2 papillary muscles. B, Fixation of the SMV to the mitral annulus using continuous sutures. C, Closing and opening state of SMV. D, Implanted state of the SMV.

**Table 1:** The design concepts of the Normo valve

• Large anterior leaflet fully covers the mitral annulus during systole • Long leg of the leaflet directly connected to 2 papillary muscles • Near normal folded structure of the commissure without any suture line • Respect the natural curve of the native anterior mitral annulus

After receiving approval from the local institutional review board (No.: 23-027; approved: May 24, 2011), we performed this operation in 2011 at our institution on a woman who wished to eventually have a baby. The goal of this study was to assess the late results of the Normo procedure.

## Patients and Methods

**Figure 2:**
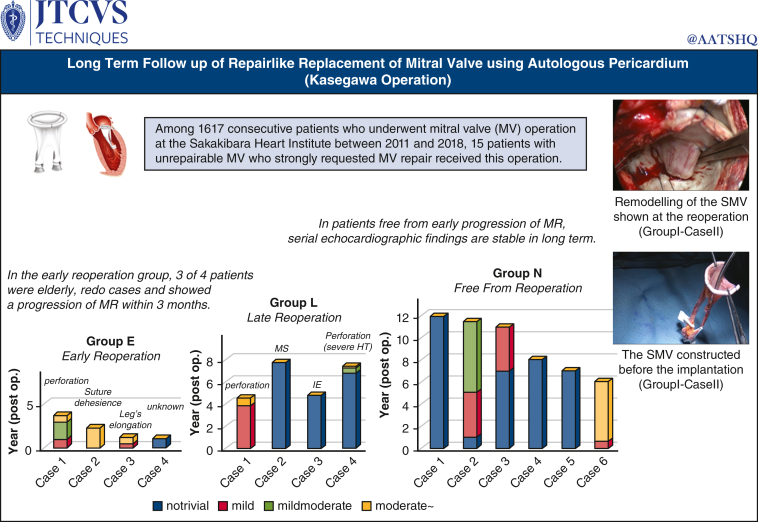
Graphical abstract. Among 1617 consecutive patients who underwent mitral valve (*MV*) surgery at the Sakakibara Heart Institute between 2011 and 2018, 15 patients with unrepairable MV who strongly requested MV repair were enrolled in this study. Although the long-term echocardiographic findings were variable, perhaps depending on the quality of the autologous pericardium used, in patients without early progression of mitral regurgitation, echocardiograph findings were stable in the long-term. *SMV*, Stentless mitral valve; *MR*, mitral regurgitation; *MS*, mitral stenosis; *IE*, infective endocarditis; *HT*, hypertention.

Between July 2011 and November 2018, 15 adult patients (9 women; mean age 48.7 ± 15.9 years) who had requested valve repair rather than replacement underwent this operation at the Sakakibara Heart Institute (Figure 2). During the same period, MV repair was performed in 969 (59.9%) of 1617 consecutive patients who underwent MV surgery at our institution. The clinical profiles of the patients are shown in Table 2. In all 15 patients who strongly requested MV repair rather than the existing MVR procedure, preoperative transthoracic echocardiography (TTE) and transesophageal echocardiography (TEE) performed in each patient revealed low feasibility for successful MV repair. This operation using AP is recommended as an alternative to MVR. The study was approved by the local institutional review board. All 15 patients agreed to undergo this surgery and provided written informed consent for publication (No.: 23-027; approved: May 24, 2011).

**Table 2:** Characteristics of the patients

No.	Age	Gender	Diagnosis	Rhythm	Etiology	NYHA functional class
1	43	F	MSR post-MVP	NSR	Rheumatic	II
2	30	M	MR	NSR	Infective endocarditis	IV
3	59	F	MSR	NSR, paf	Rheumatic	II
4	29	F	MSR post-MVP	NSR	Congenital	II
5	28	F	MSR post-MVP	NSR	Congenital	II
6	54	F	MSR	NSR	Infective endocarditis	II
7	41	M	MSR post-MVP	NSR	Rheumatic	II
8	61	M	MR post-MVP	NSR, paf	Degenerative	II
9	63	M	MR post-redo MVP	NSR, paf	Degenerative	II
10	47	M	MR post-MVP	NSR	Degenerative	II
11	69	F	MSR post-MVP	NSR, paf	Degenerative	II
12	27	F	MR	NSR	Infective endocarditis	IV
13	72	M	MR post-MVP	NSR	Infective endocarditis	II
14	66	F	MS, post-OMC	NSR, paf	Rheumatic	II
15	41	M	MR, MAC	NSR, paf	Degenerative	II

*NYHA*, New York Heart Association; *F*, female; *MSR*, mitral stenosis and regurgitation; *MVP*, mitral valve plasty; *NSR*, normal sinus rhythm; *M*, male; *MR*, mitral regurgitation; *paf*, paroxysmal atrial fibrillation; *MS*, mitral stenosis; *OMC*, open mitral commissurotomy; *MAC*, mitral annular calcification.

After discharge, patients were prospectively followed-up with periodic clinical and echocardiographic evaluations up to the end point (death or redo operation). The end point of this study was June 15, 2023.

### Operative Technique

**Figure 3:**
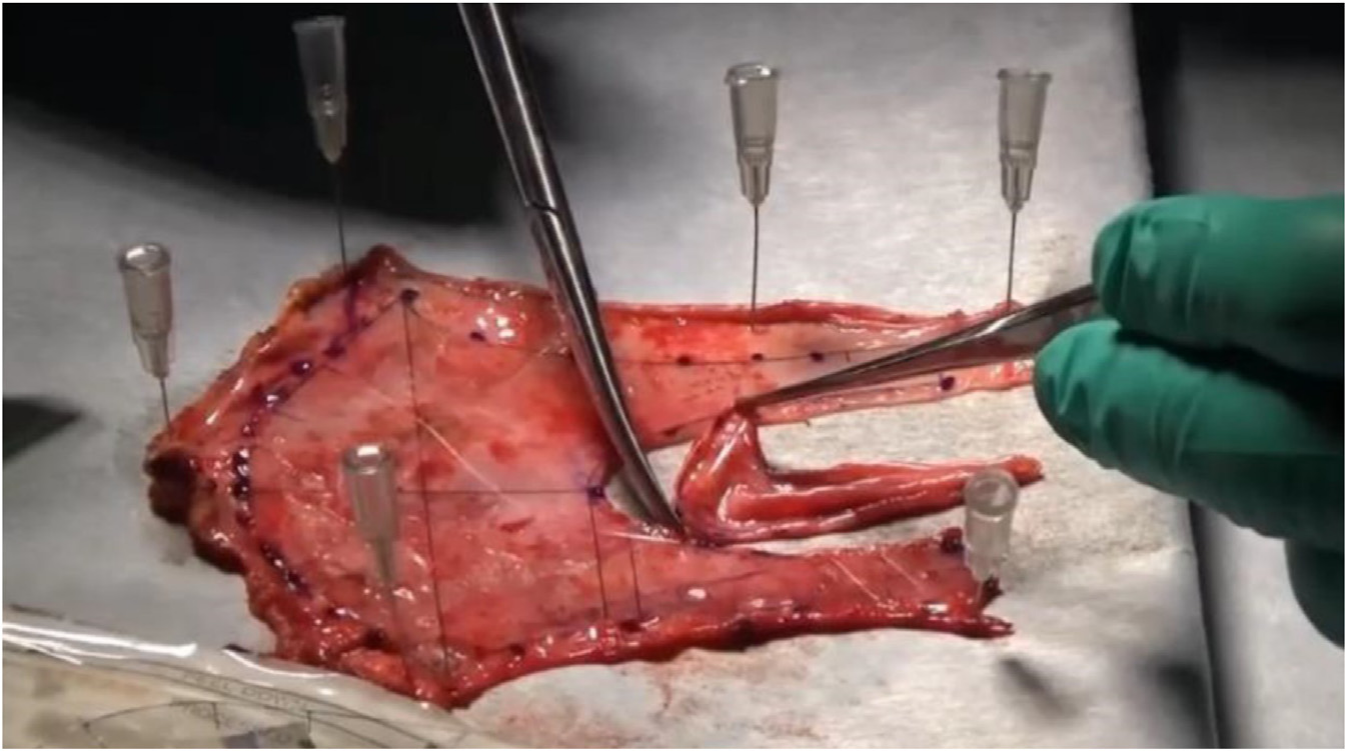
Normo valve. Fresh, nontreated autologous pericardium was formed by cutting along a template specially designed for the Normo valve.

Through median sternotomy, the AP was harvested and cut along with a specially designed template for the Normo valve, and the valve was constructed by suturing to the flexible ring or a strip of AP before cardiopulmonary bypass (Figure 3).

After confirming that the valve was not repairable, the native MV was removed, and the SMV made from nontreated AP was implanted. Figure 1 illustrates the procedure of implantation of the SMV. The distance between the plane of the mitral annulus and the fixation point on each papillary muscle was measured by placing a horizontal mattress stitch with a small pledget on each papillary muscle (posteromedial and anterolateral). The fixation point was marked on both legs of the SMV, and stitches that passed through the papillary muscle were passed through these marks on the SMV and tied down. We then assessed whether or not the length of the leg of the SMV decided by direct measurement was appropriate under a filling test of the left ventricle with a small amount of cardioplegic solution.

We confirmed good ballooning of the anterior leaflet (AL), a symmetric closure line, and performed fixation of the SMV to the mitral annulus using continuous sutures. In 2 patients in whom the flexible band was used to construct the SMV, an autologous leaflet was directly sutured to the anterior mitral annulus. In the other 2 patients, in whom a strip of AP instead of a flexible ring or band was used to construct the SMV, the autologous leaflet together with a thin strip of AP was sutured to the entire mitral annulus (Video 1).

## Results

### Early Outcomes

There were no postoperative hospital deaths or thromboembolic events. In a 30-year-old patient with severe active infective endocarditis (IE) and congestive heart failure, acute renal dysfunction occurred immediately after surgery. However, no other serious complications occurred in any of the remaining 14 patients.

### Late Survival

The mean follow-up period was 100.0 ± 35.7 months (range, 11-143 months). Late noncardiac death occurred in a 72-year-old patient with trivial MR during follow-up TTE. Ten months after the operation, he underwent an emergency abdominal operation twice for bowel obstruction at a general hospital and died of multiple organ failure. The autopsy revealed no abnormal findings in the SMV, resulting in the conclusion that there was no relationship between death and this operation. The survival rate at 5 years was 92.9% ± 6.9%.

### Findings of Echocardiography and Reoperation

Intraoperative TEE of all 15 patients revealed no or only trivial MR. Follow-up TTE was performed before discharge, at 6 months, and annually thereafter (mean, 6.2 ± 3.4 years). During the study period, 8 of 15 patients (53.3%) underwent reoperation after surgery (1 to 8 years after the operation). The postoperative courses of 14 patients, excluding 1 patient of late noncardiac death, were divided into 3 groups depending on the occurrence of redo surgery: Group E, early reoperation group (reoperation within 4 years; n = 4; age 45.0 ± 14.6 years); Group L, late reoperation group (reoperation after 4 years; n = 4; age 43.3 ± 16.7 years); and Group N, no reoperation group (n = 6; age 40.0 ± 17.9 years).

**Figure 4:**
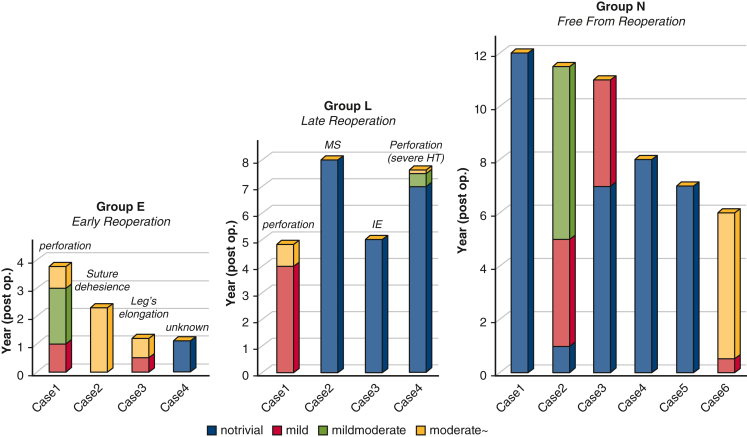
Serial echocardiographic change in mitral regurgitation (*MR*). Patients were divided into 3 groups depending on the occurrence of redo surgery: Group E, early reoperation (<4 years [n = 4]); Group L, late reoperation (>4 years [n = 4]); and Group N, no reoperation (n = 6). In group E, 3 of 4 patients experienced progression of MR within 3 months and received redo surgery in the early stage. Patients who did not show MR progression in the early postoperative stage were stable over the long-term. *MS*, Mitral stenosis; *IE*, infective endocarditis; *HT*, hypertention.

In 3 patients in Group E, the progression of MR was detected within 3 months. In Group L, acute IE in 1 patient, perforation of the leaflet in 2 patients, and sclerotic change of the leaflet in 1 patient were considered causes of valve failure. In Group N, the latest TTE examination, performed 7.2 ± 2.9 years (range, 6 to 11.5 years) after the operation, revealed no MR in 2 patients, mild MR in 2 patients, mild-to-moderate MR in 1 patient, and moderate MR in 1 patient; these 6 patients had been living normal daily lives (New York Heart Association functional class I or II) without taking Coumadin. Figure 4 shows the change in MR findings after discharge. As indicated in Figure 4, MR was well controlled, even when it became more than mild. Follow-up TTE of a 30-year-old man revealed mild to moderate 5 years after the operation. However, the MR of this patient involved 3 leakages and has remained stable since then. These 6 patients had been living normal daily lives (New York Heart Association functional class I or II) without taking Coumadin. Table 3 shows the changes in the mean mitral pressure gradient (PG).

**Table 3:** Serial echocardiographic changes in mean transmitral presssure gradient

Period	At discharge	1 y	3 y	5 y	7 y
PG (mm Hg)	4.3 ± 1.7	5.4 ± 2.3	6.0 ± 2.8	5.5 ± 3.0	8.7 ± 3.9
N	14	14	8	8	5

Values are presented as mean ± SD unless otherwise noted. *PG*, Pressure gradient.

**Figure 5:**
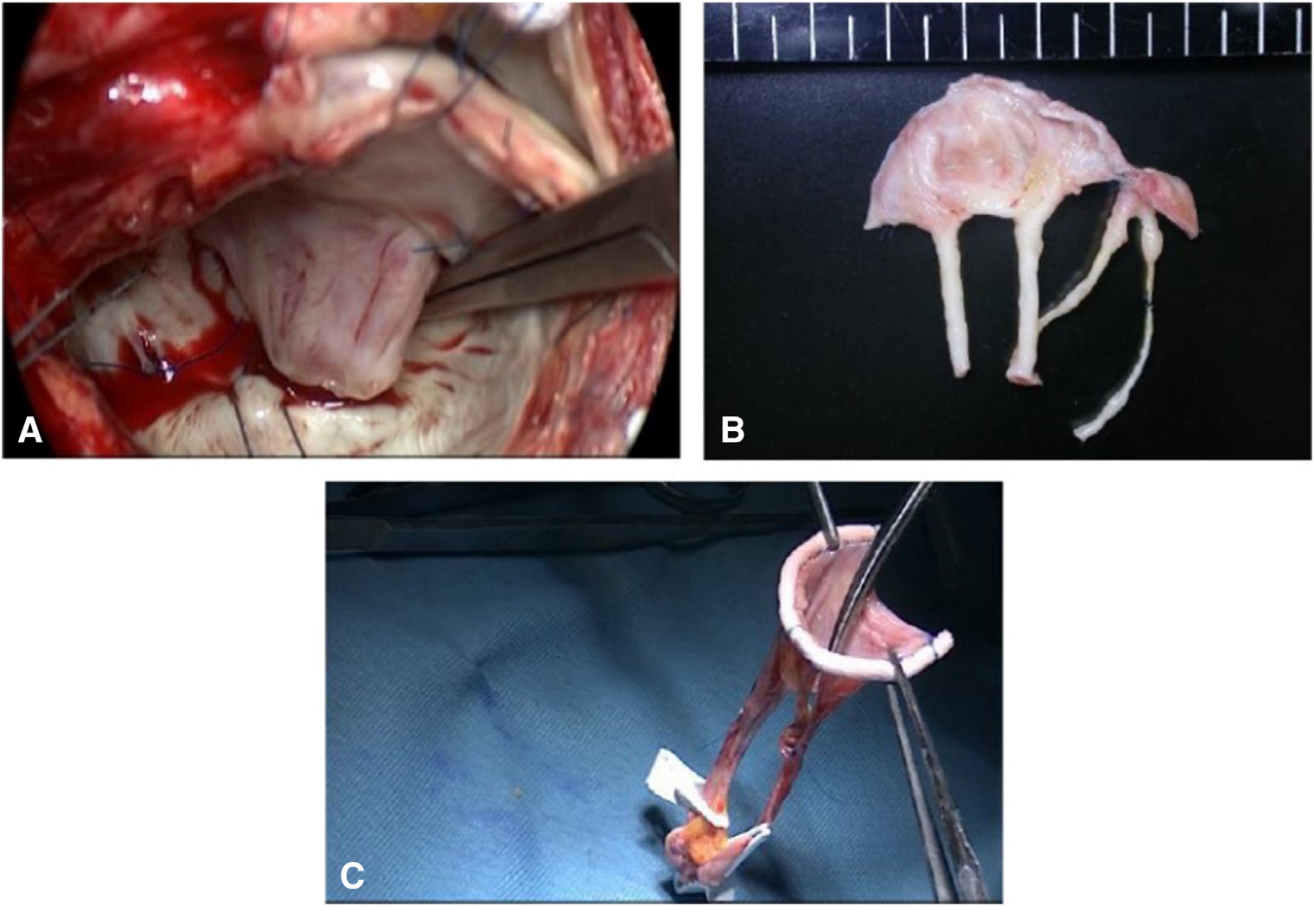
Intraoperative findings of redo mitral valve replacement (*MVR*). Intraoperative findings of redo MVR (A and B) performed 14 months after the operation and the stentless mitral valve (*SMV*) constructed during this operation. A, The configuration of the anterior leaflet (*AL*) was similar to that of the normal MV structure, including a rough zone and a clear zone, and the surface of the thin AL was entirely covered with endothelium. B, The excised SMV shows remodeling of the leg of the valve. The medial leg was elongated. C, The SMV before implantation shows a thin and narrow leg on the medial side.

Among 8 patients who received reoperation after this operation, the cause of failure was unclear in only 1 patient. This 41-year-old patient, in Group E, lived abroad before and after the surgery. Thirteen months after the operation, he underwent emergency MVR abroad for acute MR. Just 1 month before this event, he came to our institute for a 1-year postoperative examination, and the echocardiography-Doppler study revealed good performance of the valve with only trivial MR and a low PG (2.5 mm Hg). Unfortunately, we were unable to identify the exact etiology of acute MR based on the information from the hospital, although the possibility of endocarditis cannot be discounted. The other 3 patients in Group E were older than age 60 years and needed dissection of the adhesion of the AP due to previous MV repair. Figure 5 shows the intraoperative findings of redo MVR performed 14 months after this operation in a 69-year-old patient (Group E, Case 3). In these 3 patients, MR progressed within 3 months.

In Group L, active IE occurred in a young woman immediately after she became pregnant. Perforation of the AL near the large vegetation was found during the reoperation. It was considered the cause of structural valve deterioration (SVD) in 2 patients. In the remaining 1 patient, uncontrolled hypertension was a major concern at the outpatient clinic. In a 58-year-old patient with rheumatic heart disease, a reduction in the size of the huge mitral annulus resulted in several paravalvular leakages during the operation, and the technique of covering the posterior mitral annulus with a large piece of AP was considered the cause of the high-pressure gradient at the early stage. Progression of mitral stenosis (MS) was the cause of SVD in this patient. The excised SMV of the patient revealed thickening of the autologous leaflet only in the posterior side where the additional procedure was performed. There were no remarkable changes on the AL nor on the legs of the SMV.

## Discussion

The Normo operation consists of the construction of the SMV using AP (like MV repair) before the institution of cardiopulmonary bypass and the implantation of the valve (like replacement).^6^ This is why we call this operation a repair-like replacement. This study reported the late results of Normo valve implantation. Despite excellent early results, the incidence of reoperation is high during the postoperative period. However, the patients who did not show progression of MR in the early postoperative stage were stable in the long-term.

To overcome the disadvantages of MVR, Frater and colleagues^7^ developed a chordal-supported SMV (Quattro; St Jude Medical) in 1994 made from glutaraldehyde-treated bovine pericardium and demonstrated annulo-papillary continuity. The Quattro valve has been used in a limited number of institutions, and good early and midterm results have been reported.^7^, ^8^, ^9^, ^10^, ^11^, ^12^ However, the long-term durability remains unclear. What differentiates the Normo valve from the Quattro valve is that the Normo valve is completely flexible, made from the nontreated patient’s own tissue, composed of 2 leaflets, and anatomically similar to the normal MV.^5^ The advantages of the Normo operation are as follows: the valve can be constructed with a clear view without any time limit before the initiation of cardiopulmonary bypass, the competency of the valve can be confirmed following its construction, and the technique of implanting the valve is very simple and reproducible. These advantages are considered to have resulted in the excellent intraoperative TEE findings of the patients in this study. However, the major disadvantage of this operation is the variableness of the AP used to make the valve. It takes time to harvest the patient’s AP, especially in patients with a history of previous cardiac surgery. In such patients, the AP can be thickened, and the technique for dissecting the adhesion of the AP can cause microinjury, subsequently leading to perforation of the leaflet of this valve.

In this study, despite excellent early results, 8 of 15 patients underwent reoperation during postoperative period. The cause of reoperation was SVD in 7 patients (MR in 6, MS in 1) and non-SVD (ie, IE) in 1. Excluding 1 patient who received emergency MVR abroad, the starting point of SVD detected by an echocardiography-Doppler examination was a small perforation of the AL near the suture line in 3 patients, dehiscence of the interleaflet stitch in 1 patient, elongation of 1 leg of the posterior leaflet in 1 patient, and MS possibly due to an additional surgical procedure in 1 patient. In the early reoperation group, 3 of the 4 patients had MR progression within 3 months. All 3 of these patients were elderly (older than age 60 years) and had a history of MV repair, which consequently indicates a low quality of the AP. SMV remodeling is considered to occur several months after the operation. Remodeling, including transformation of the leg from a strip-like structure to a roll-like structure (Figure 5), is considered to strengthen the component of the SMV. We believe that afterload reduction is indispensable in the early stage because Normo valves with only 2 pairs of legs are structurally weak before remodeling completion. The results of changes in MR (Figure 4) demonstrated that patients who did not show progression of MR during the early postoperative stage were stable in the long-term.

Excellent early results are considered to correspond with the results of simulator testing and animal experimental studies.^5^^,^^13^ The stability of the valve competency shown in the results of the no reoperation group is considered to reflect the design of the Normo valve. The deep zone of coaptation of the Normo valve ensures good competency even when the leg length is less than optimal. Simulator and animal studies^13^ have demonstrated an excellent diastolic function of the Normo valve. However, in a clinical study,^14^ a postoperative echocardiography examination showed a higher mean PG than that observed after conventional MV repair. This might have been a reflection of the quality of the AP used. The postoperative mean PG of the patient using a large band (33 mm) instead of a ring remained under 5 mm Hg at 8 years after the operation, and that of 1 patient without a ring/band remained at 3.9 mm Hg at 6 years after the operation. We believe that the cause of the high PG is not the design of the valve but the quality of the AP. As previously described, the opening function of the Normo valve was evaluated repeatedly using mechanical simulator testing. In terms of generating identical testing conditions, an evaluation of the hydrodynamic function of prostheses using a simulator is far superior to the use of either clinical or animal experimental studies, with very high reproducibility of the results.

After submitting the results of simulator testing, an animal experimental study, and 14 multicenter clinical studies performed at 5 institutes in Japan between 2011 and 2015, this operation was approved by the government as an advanced medical treatment in December 2015, and clinical testing was started in January 2016. However, it was withdrawn after being performed in 6 patients, including the 4 of this study. In the present study, 10 (67%) of 15 patients had a history of MV repair, and 5 patients (30%) were older than age 60 years. We therefore considered that using the AP of elderly patients or patients with a history of MV repair is associated with a high risk of SVD of the SMV.

### Limitations

This study represents the long-term follow-up of a newly developed repair-like replacement of MV. Because the number of treated patients was limited and no control group was available, statistical analyses were not performed.

## Conclusions

Despite excellent early results, the incidence of reoperation is high during the postoperative period. Although the cause of the high incidence of valve failure is not clear, it appears to be related to the fact that patients in this study included many elderly and redo patients. By respecting the quality of the autologous pericardium, the Normo operation can be a viable alternative to valve replacement, especially for young patients with an unrepairable valve who wish to avoid conventional MVR.

## Conflict of Interest Statement

The authors reported no conflicts of interest.

The *Journal* policy requires editors and reviewers to disclose conflicts of interest and to decline handling or reviewing manuscripts for which they may have a conflict of interest. The editors and reviewers of this article have no conflicts of interest.

## Supplementary Data

Video 1 Normo valve operation. In this case of active infective endocarditis in a 27-year-old woman, preoperative echocardiography revealed low feasibility of successful mitral valve repair. Because the patient strongly wanted to avoid a conventional mitral valve replacement, we recommended this operation and she agreed. The Nomo valve as made from fresh autologous pericardium and a flexible ring was implanted. Follow-up echocardiograph taken at the outpatient clinic 7 years after the operation still showed a competent valve. Video available at: https://www.jtcvs.org/article/S2666-2507(24)00076-2/fulltext.
