# Supplementary figures and images for: Long-term follow-up of repair-like replacement of mitral valve using autologous pericardium
Source: JTCVS Tech. 2024 Mar 6;25:55–62. doi: 10.1016/j.xjtc.2024.02.020 (PMC11184484; doi:10.1016/j.xjtc.2024.02.020)

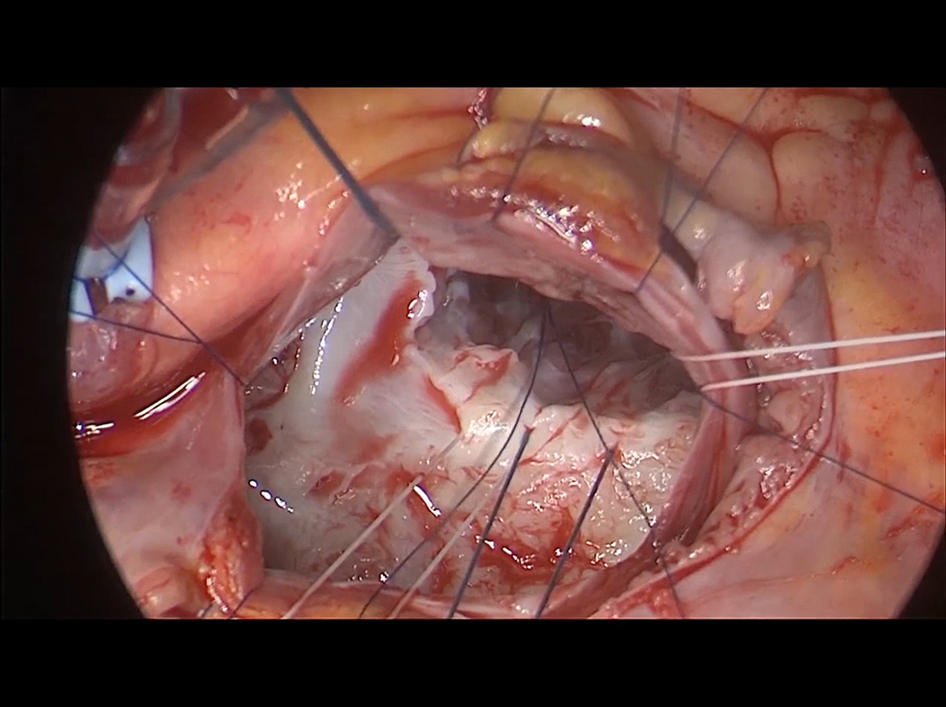

Supplement: Video 1 — Normo valve operation. In this case of active infective endocarditis in a 27-year-old woman, preoperative echocardiography revealed low feasibility of successful mitral valve repair. Because the patient strongly wanted to avoid a conventional mitral valve replacement, we recommended this operation and she agreed. The Nomo valve as made from fresh autologous pericardium and a flexible ring was implanted. Follow-up echocardiograph taken at the outpatient clinic 7 years after the operation still showed a competent valve. Video available at: https://www.jtcvs.org/article/S2666-2507(24)00076-2/fulltext. [file fx2.jpg]
